# The functional outcomes and complications of different reconstruction methods for Giant cell tumor of the distal radius: comparison of Osteoarticular allograft and three-dimensional-printed prosthesis

**DOI:** 10.1186/s12891-020-3084-0

**Published:** 2020-02-03

**Authors:** Yitian Wang, Li Min, Minxun Lu, Yong Zhou, Jie Wang, Yuqi Zhang, Xinzhu Yu, Fan Tang, Yi Luo, Hong Duan, Chongqi Tu

**Affiliations:** Department of Orthopedics, West China Hospital, Sichuan University, No. 37 Guoxuexiang, Chengdu, 610041 Sichuan People’s Republic of China

**Keywords:** Distal radius, Giant cell tumor, Osteoarticular allograft, 3D-printed prosthesis

## Abstract

**Background:**

En bloc excision has been increasingly used for the management of giant cell tumors (GCTs) in the distal radius. An osteoarticular allograft has been used extensively for decades, and custom-made prosthesis reconstruction has been more recently applied. We aimed to compare the clinical outcomes of the two procedures.

**Methods:**

We retrospectively analyzed 30 patients with Campanacci III or recurrent GCTs of the distal radius for follow-up at a mean of 33.2 months. In total, 15 underwent osteoarticular allograft reconstruction (allograft group) and 15 received cementless three-dimensional (3D)-printed prosthesis reconstruction (prosthesis group) between March 18, 2013, and May 20, 2018. All patients underwent by clinical and radiological examinations, including pre- and postoperative active range of motion (ROM) of the wrist, VAS score, grip strength, degenerative change of wrist, Mayo wrist score and Musculoskeletal Tumor Society (MSTS) score. Complications were evaluated using the Henderson classification.

**Results:**

Both groups showed significantly increased ROM, grip strength, Mayo score and MSTS score postoperatively. Furthermore, the extension, flexion, MSTS, and Mayo score were significantly higher in the prosthesis group. There was no significant difference in grip strength and VAS between the groups. In allograft group, one patient had a late infection one had resorption of allograft without allograft bone fracture. and four had wrist subluxation. All patients had degenerative changes (mean 9 months). In the prosthesis group, three patients developed wrist subluxation, three had separation of the distal radioulnar joint, and none of the patients developed wrist degeneration.

**Conclusions:**

Our study compared the objective functional outcomes and complications of two reconstructive methods for Campanacci III or recurrent GCT in the distal radius. 3D-printed prosthesis replacement can partially preserve wrist function better than allograft reconstruction in the short-term. During the design of 3D-printed prosthesis, preoperative morphological assessment of the affected proximal row carpal is helpful to control postoperative dislocation. After allograft reconstruction, wrist degeneration, which has been demonstrated in all patients, severely influence their wrist function. Therefore, compared to allograft reconstruction, 3D-printed prosthesis reconstruction has irreplaceable advantages at early-stage application, especially in wrist function, however, further studied with a larger number of cases and longer follow-up.

## Introduction

The distal radius is the third most common location for giant cell tumor (GCT) after the distal femur and proximal tibia, and approximately 10% of GCT involve the distal radius [1, 2]. Although the majority of studies do not support the theory that GCT in the distal radius are more aggressive, controversy exists on the surgical options for patients with GCT in the distal radius, as well as it’s the rate of recurrence. Intralesional curettage and cement packing is the most common treatment for Campanacci I and II lesions [3, 4]. However, for Campanacci III or recurrent GCT of the distal radius, en bloc resection and reconstruction is recommended; this is associated with a lower risk of local recurrence and poorer functional outcomes than intralesional surgery [3, 5–7]. Reconstruction of the wrist joint following en bloc resection of the distal radius is challenging because of the high functional demands of the wrist, limited surrounding soft tissue, limited bone mass and the proximity of important nerves and tendons [8, 9]. Numerous reconstructive procedures have been described including prosthetic replacement [9–11], osteoarticular allograft [12, 13], allograft fusion [14], arthrodesis using bulk autograft [1, 2], ulnar translocation [15], and non-vascularized [16] or vascularized [17] fibular graft with or without arthrodesis [18]. Although these techniques have unique advantages and inevitable complications, a gold standard for distal wrist reconstruction has not yet been established.

As a major treatment method, osteoarticular allografts have advantages, including osteoinduction, use as a biologic scaffold, and generally appropriate anatomical match for host proximal row carpel [13, 19]. However, the use of osteoarticular allografts is extremely restricted by the limited quantity and severe complications, including ankylosis, rejection, and allograft fracture [19]. As a result, several prosthetic arthroplasties have been reported in the last decade, and acceptable results of prosthesis reconstruction were demonstrated short- to mid-term [9, 20, 21]. However, potential complications included aseptic loosening caused by cement-fixation and inappropriate anatomical matching [11, 22]. Following technological advances in materialogy and manufacturing, 3-dimensional (3D) printing technology has been introduced in the field of orthopedics. Our institution reported the first study on a custom-made cementless 3D-printed prosthesis for distal radius GCTs [21]. No reports regarding the comparative study of osteoarticular allograft and prosthetic replacement has been published previously. The purpose of this study was to investigate the mid-term clinical outcomes of patients with GCT in the distal radius after these two reconstructive methods.

## Methods

### Patients

In total, 30 patients, who underwent en bloc resection of Campanacci III or recurrent GCT in the distal radius and allograft or prosthesis reconstruction between March 18,2013, and May 20, 2018, were enrolled in our study. The histopathologic diagnosis for each patient was obtained by needle biopsy. In order to determine the reconstructive option for each subject, the surgical indication was based on patient’s preference. The patients, who selected biological reconstruction and rejected arthrodesis using autograft and/or ulnar translocation, underwent osteoarticular allograft. Whereas, patients, who selected endoprosthetic reconstruction and rejected arthrodesis using autograft and/or ulnar translocation underwent 3D-printed prosthesis replacement. We excluded patients with metastasis and those who had any surgical procedure unrelated for Campanacci III or recurrent GCT in the distal radius. According to the reconstructive methods, we considered a consecutive series in our institution with GCTs of the distal radius treated with osteoarticular allograft (allograft group) and 3D-printed prosthesis (prosthesis group). Preoperative assessments included radiographs of the bilateral forearm and wrist, computed tomography (CT) scan of the chest, bilateral forearm and wrist, magnetic resonance imaging (MRI) of the affected side and an optional total body bone scan. All patients were evaluated for pain according to a 10-cm VAS score, range of motion (ROM) recorded using a goniometer, grip strength of bilateral wrist joint, Mayo wrist score [23] and Musculoskeletal Tumor Rating Scale (MSTS) [24] of the wrist, preoperatively and postoperatively. All patients were assessed every 3 months during the first year of follow-up and every 6 months thereafter with a physical examination, VAS, functional evaluation of the wrist, radiographs of the wrist and chest. The degenerative changes of the wrist were evaluated radiographically according to Knirk and Jupiter scale in both groups [25]. This study was performed according to the principles embodied in the Declaration of Helsinki and the Institutional Review Board of Sichuan University West China Hospital. Written informed consent was obtained from all patients when they began treatment for osteoarticular allograft or 3D-printed prosthesis.

#### Allograft preparation and prosthesis design

In the allograft group, to obtain a reconstruction as anatomical as possible, the isometric x-rays of the bilateral side and a 3D-CT scan were taken. Fresh-frozen allografts were supplied by the bone-bank facility (West China Hospital, Chengdu, Sichuan) without soft tissue.

**Fig. 1:**
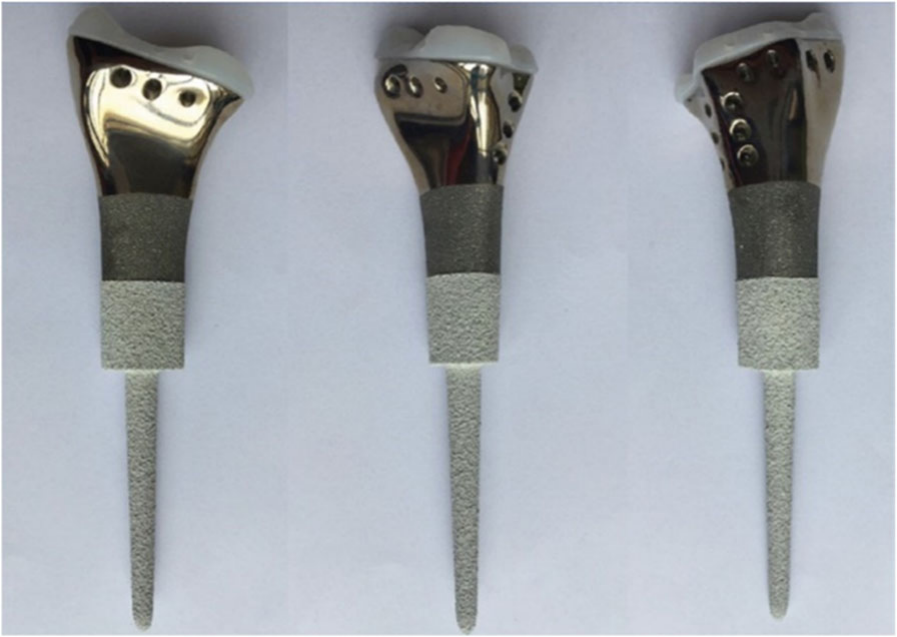
Prosthesis is composed of polyethylene, repairing pores, shaft and stem coated with hydroxyapatite

In the prosthesis group, all prostheses were custom-made for each patient by our team and produced (Chunli, Beijing, People’s Republic of China). Based on our previous study [21] and experience, the design was modified and improved with the help of Mimics V17.0 software (Materialise Corp., Leuven, Belgium). The main components of the 3D-printed prosthesis were an ultrahigh-molecular-weight polyethylene (Orthoplastics Ltd., Lancashire, UK), repairing pores for soft tissue reconstruction, shaft and stem coated with hydroxyapatite (titanium alloy) (Fig. 1). All prostheses were printed by electron beam melting technology (ARCAM Q10, Mölndal, Sweden). It took 2 or 4 weeks to manufacture the prosthesis, during which time the patients were treated with NSAIDs if necessary.

#### Surgical technique and postoperative management

Patients were submitted to general anesthesia. The tumor was resected en bloc through a dorsal approach including the previous biopsy and operating incision. Soft tissue dissection, including the wrist ligaments, capsule, triangular fibrocartilage complex (TFCC), biopsy track and hematoma, was dependent on the tumor border. A step-cut osteotomy was performed with a safe margin (1.5 cm from the tumor border) according to X-rays and MRI. In the allograft group, an appropriate locking compression plate (LCP) was fixed to the allograft bone and the remaining radius to obtain initial stability and avoid rotation of the distal radius Allograft bone was fixed with a suitable LCP, Wego, Shandong, People’s Republic of China) to bridge the allograft and the remaining radius [13]. In the prosthesis group, stable fixation between the prosthesis and the reamed radius canal was enhanced by press-fitting the distal stem. The remaining dorsal and/or palmar ligaments, joint capsule and TFCC were sutured to the allograft bone or repairing pores of the prosthesis.

Based on the reserved soft tissue, an above-elbow cast was administered to patients with massive resection. After a 4-week immobilization, active wrist exercises were acceptable.

#### Statistical analysis

Survival data were compiled using Kaplan-Meier analysis. Prosthesis survivorship was determined for implants using revision or removal of the components for any reason as an end point, according to Henderson et al. [26]. No complication survivorship was defined for patients without wrist pain, degeneration of the wrist, subluxation, and separation of the distal radioulnar joint at the last follow-up.

The normality of the continuous data was verified by the Shapiro-Wilk test. Normally and abnormally distributed parameters were assessed by the independent sample t-test and the Mann-Whitney U test, respectively. A *P*-value of < 0.05 was determined to be statistically significant. Comparisons were conducted between the allograft and prosthesis group by log-rank test. Data analyses were performed using SPSS 20.0 software (IBM Corporation, Armonk, NY, USA).

## Results

### Patient characteristics

In total, 30 consecutive patients were enrolled from March 18, 2013 to May 20, 2018. All pathology materials were confirmed at West China Hospital. The clinical characteristics of the two groups and their comparison are presented in Table 1. Patients of the two treatment groups had no significant difference in gender, age, and follow-up time.

**Table 1:** Demographic and radiographic characteristics of the two treatment groups

Characteristic	Osteoarticular allograft group			3D-printed prosthesis group			p-value
	Mean	SD	Range	Mean	SD	Range	
Number of patients	15	–	–	15	–	–	–
Male/female	8/7	–	–	6/9	–	–	0.50
Age (years)	37.3	12.1	24–63	38.0	10.0	21–56	0.87
Side (right/left)	7/8	–	–	8/7	–	–	0.67
Primary/Recurrence	11/4	–	–	10/5	–	–	0.7
Length of resection (cm)	8.0	1.0	7–10	5.4	1.7	3.5–9	< 0.001
Follow-up (months)	34.4	16.8	10.7–64.4	31.4	11.8	13.7–48.2	0.52

### Functional outcomes

There was no significant difference between the two groups in preoperative functional outcomes. In the allograft group, there was a significant increase in ROM postoperatively, with exception of flexion. The mean postoperative ROM of the wrist, was 38.3 ° active extension (range, 25 ° 65 °, *P* < 0.01), 26.3 ° flexion (range, 10°-50 °, *P* = 0.65), 52.0 ° pronation (range, 30°-65°, *P* = 0.04), and 55.0 ° supination (range, 40 °-80 °, *P* = 0.03). The MSTS wrist score and Mayo wrist score were significantly increased postoperatively, averaging 71.2% (57.1–82.9%, P = 0.03) and 65.0 (55–75, *P* < 0.01) respectively. Postoperative grip strength was 54.7% (range, 42.5–70.3%) of the normal contralateral hand compared to 29.7% (range, 15.9–38.3%) preoperatively, with a significant increase(P < 0.01). The VAS score was 5.5 (range, 4–7) preoperatively and 1.2 (range, 0–3) postoperatively with significant difference(*P* = 0.05) (Table 2).

**Table 2:** preoperative/postoperative details in allograft group

Patient number	Age (years)/gender	Campanacci grade	Follow-up (months)	Range of motion				Grip strength(% of normal side)(%)	VAS score	Degenerative grade/ Occurrence time after operation (month)	Mayo score	MSTS							
				Extension (°)	Flexion (°)	Supination (°)	Pronation (°)					total	motion	Extension (°)	Flexion (°)	Supination (°)	Pronation (°)	Functional Activity	Emotional Acceptance
1	40/M	III	64.4	30/30	20/25	30/55	40/40	33.2/63.0	6/0	III/9	20/65	48.6/77.1	5/5	1/5	5/5	5/5	1/3	1/3	1/5
2	38/M	Recurrent	61.8	40/65	25/30	35/55	40/55	18.7/60.2	5/2	III/12	25/65	60.0/65.7	5/5	1/5	5/5	3/5	1/3	1/3	5/3
3	33/M	Recurrent	47	20/25	30/20	25/50	35/40	32.6/42.5	7/1	III/6	15/55	48.6/57.1	3/5	1/5	5/5	5/3	1/3	1/0	1/1
4	25/M	III	38.2	35/65	30/50	30/80	35/65	15.9/63.3	5/1	III/12	10/75	60.0/82.9	5/5	1/5	5/5	5/5	1/3	3/3	1/5
5	29/M	Recurrent	50	35/65	40/25	20/65	25/55	24.6/70.3	6/1	III/12	15/75	48.6/71.4	5/5	1/5	5/5	5/5	1/3	3/3	1/3
6	25/M	III	13.1	20/25	10/20	20/40	15/55	37.9/61.7	6/0	III/3	20/70	60.0/65.7	3/5	1/5	5/5	5/5	3/3	3/1	1/1
7	62/F	III	10.7	35/30	40/35	35/60	40/60	28.2/45.8	5/1	III/12	20/65	54.3/77.1	5/5	1/5	5/5	5/5	1/3	1/3	1/3
8	37/F	III	27.9	45/25	45/25	45/45	45/65	34.5/61.3	6/1	III/6	20/70	54.3/77.1	5/5	1/5	5/5	5/5	1/3	3/3	3/3
9	35/M	III	24.5	20/25	15/35	35/50	25/55	37.9/51.1	4/1	III/9	15/60	60.0/65.7	3/5	1/5	5/5	5/5	3/3	3/3	1/3
10	27/F	III	22.5	30/30	25/20	40/45	40/45	38.0/62.3	5/1	III/12	20/65	60.0/71.4	5/5	1/5	5/5	5/5	1/3	3/3	1/3
11	63/F	III	20.9	20/25	20/10	30/45	30/45	28.1/46.4	5/3	III/6	15/55	42.9/71.4	3/5	1/5	5/3	5/3	1/3	3/3	1/3
12	24/F	III	18	25/30	25/25	35/55	40/35	28.1/44.2	6/2	III/8	20/60	65.7/71.4	5/5	1/5	5/5	5/5	3/3	1/3	3/3
13	35/F	Recurrent	43	25/40	5/25	20/50	35/55	28.1/47.2	7/2	III/12	15/60	45.7/71.4	3/5	0/5	5/5	5/5	3/3	1/3	1/3
14	45/M	III	42	30/50	25/25	50/65	35/50	21.3/46.5	4/1	II/9	15/65	54.3/77.1	5/5	3/5	5/5	5/5	1/3	1/3	1/3
15	42/F	III	32	35/45	20/25	45/65	30/60	38.3/55.6	6/1	II/12	20/70	48.6/65.7	5/5	1/5	5/5	5/5	1/3	1/3	1/1

**Fig. 2:**
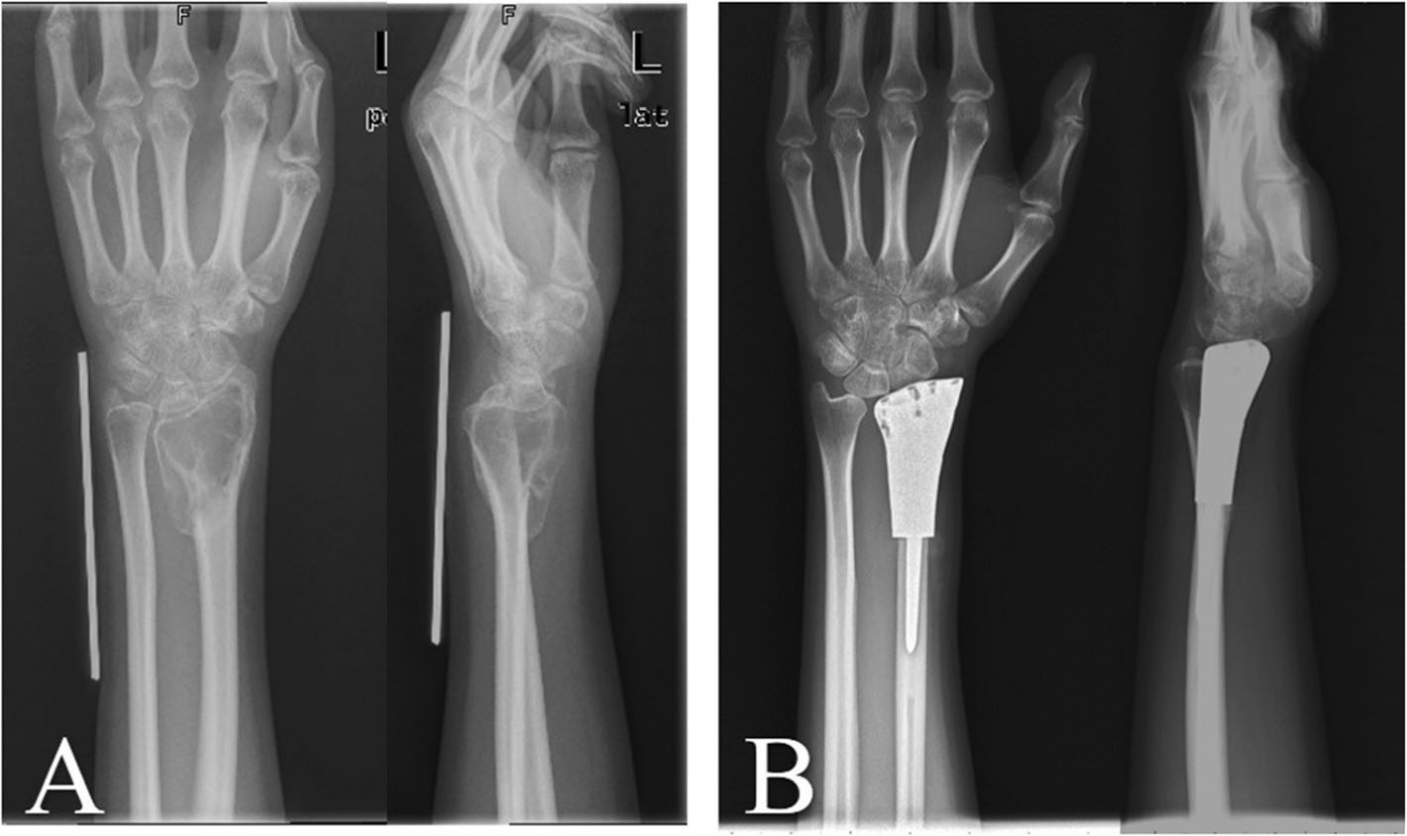
**a**: The Campanacci III GCT of left distal radius was diagnosed; **b**: 10 months after surgery

In the prosthesis group, with regards to all aspects of the ROM, there were significant differences between the pre- and post-operative measurements. After prosthesis reconstruction there was a 61.7 ° active extension (range, 20 °-85 °, *P* < 0.01), 45.0 ° flexion (range, 20 °-80 °, *P* = 0.04), 54.7 ° pronation (range, 30 °-80 °, *P* < 0.01), and 60.0 ° supination (range, 25 °-85 °, P < 0.01) (Fig. 2). With respect to the MSTS score of the wrist and the Mayo wrist score, there was a significant increase after the operation of 81.7% (60–94.3%, *P* < 0.01) and 71.0 (40–85, P < 0.01) on average, respectively. There were significant differences between the pre- and post-operatively in grip strength. There were 33.2% (range, 12.8–62.7%, *P* < 0.01) and 64.4% (range, 31.9–100%, *P* < 0.01) respectively. Furthermore, there was a significant difference in VAS score, which was 5.5 (range, 4–7) preoperatively and 1.3 (range, 0–4) postoperatively(P < 0.01). (Table 3).

**Table 3:** preoperative/postoperative details in prosthesis group

Patient number	Age (years)/gender	Campanacci grade	Follow-up (months)	Range of motion				Grip strength(% of normal side)(%)	VAS score	Degenerative grade/ Occurrence time after operation (month)	Mayo score	MSTS							
				Extension (°)	Flexion (°)	Supination (°)	Pronation (°)					Total	motion	Pain	Stability	Deformity	Strength	Functional Activity	Emotional Acceptance
1	42/F	III	48.2	25/45	40/40	25/75	40/80	12.8/31.9	6/0	None	30/60	31.4/88.6	3/5	1/5	3/5	3/5	0/3	0/3	1/5
2	45/M	Recurrent	45.7	45/85	25/35	50/80	30/50	28.3/45.2	5/2	None	20/70	51.4/77.1	5/5	1/3	3/3	5/5	3/3	1/3	0/5
3	34/F	III	45.3	40/60	30/40	45/60	30/45	21.3/46.8	6/1	None	15/75	45.7/88.6	5/5	1/5	3/3	5/5	3/3	1/5	0/5
4	37/M	Recurrent	35.4	10/60	30/45	20/50	30/50	34.0/54.3	7/1	None	15/75	51.4/82.9	3/5	1/5	5/3	5/5	3/3	1/5	0/3
5	45/F	III	34.7	50/65	70/80	20/50	30/50	42.6/73.9	5/1	None	20/75	57.1/82.9	5/5	1/5	5/3	5/5	3/3	1/3	0/5
6	46/M	Recurrent	33.8	30/80	40/60	20/50	30/50	35.9/85.0	6/1	None	15/75	57.1/82.9	5/5	1/3	5/3	5/5	3/3	1/5	0/5
7	37/F	III	31.7	50/75	40/50	50/60	45/55	32.2/81.5	5/1	None	20/80	57.1/82.9	5/5	1/5	5/3	5/3	3/3	1/5	0/5
8	27/F	III	30.7	60/75	60/60	50/65	50/70	22.9/56.7	6/1	None	25/75	54.3/88.6	5/5	1/5	5/5	5/5	1/3	1/3	1/5
9	24/F	III	26.3	15/50	10/45	30/60	20/60	38.3/76.7	4/1	None	15/75	51.4/82.9	3/5	3/5	5/3	5/5	1/3	1/3	0/5
10	21/F	III	17.9	30/85	30/30	45/85	45/55	18.4/100.0	5/1	None	30/85	51.4/94.3	5/5	1/5	5/5	5/5	1/5	1/5	0/3
11	45/M	III	42.4	15/20	20/20	20/25	20/30	41.8/53.2	5/4	None	15/40	62.9/60.0	3/3	3/3	5/3	5/1	3/3	3/3	0/5
12	45/F	III	36.7	20/70	30/50	45/60	30/35	39.0/64.6	6/2	None	15/75	57.1/82.9	5/5	1/3	5/5	5/5	3/3	1/3	0/5
13	56/M	Recurrent	14.4	10/55	5/40	20/60	30/60	37.8/56.7	7/2	None	10/70	54.3/88.6	3/5	0/5	5/5	5/5	3/3	3/3	0/5
14	25/F	III	14.4	50/65	40/50	50/70	40/60	30.4/56.7	4/1	None	20/75	57.1/82.9	5/5	3/5	3/5	5/5	3/3	1/3	0/3
15	41/M	Recurrent	13.7	30/35	25/30	50/50	40/50	62.7/83.6	6/1	None	25/60	57.1/60.0	5/5	1/5	5/3	5/3	3/3	1/1	0/1

With respect to the postoperative functional outcomes, the extension (38.3 vs 61.6, P < 0.01), flexion (26.3 vs 45.0, *P* = 0.02), grip strength (54.7 vs 64.4, *P* = 0.03), MSTS score (71.2 vs 81.7, *P* = 0.01), and Mayo score (65.0 vs 71.0, P = 0.01) were significantly higher in the prosthesis group. There was no significant difference in supination, pronation, and VAS score between the two groups (Table 4).

**Table 4:** Statistical comparison of clinical results between the two treatment groups

Measure	Osteoarticular allograft group (*N* = 15)	3D-printed prosthesis group (N = 15)	p-value allograft vs prosthesis
Postoperative			
Extension (°)	38.3 (range 25~65)	61.7(range 20~85)	< 0.01
Flexion (°)	26.3 (range 10~50)	45.0 (range 20~80)	0.02
Supination (°)	55.0 (range 40~80)	60.0(range 25~85)	0.187
Pronation (°)	52.0 (range 30~65)	54.7 (range 30~80)	0.683
Mayo score	65.0 (range 55~75)	71.0 (40~85)	0.013
MSTS score	71.2 (range 57.1~82.9)	81.7 (range 60~94.3)	0.01
VAS score	1.2 (range 0~3)	1.3 (range 0~4)	0.806
Grip strength (% of normal side) (%)	54.7 (range 42.5~70.3)	64.4 (range 31.9~100)	0.03
The variations before and after surgery			
Extension (°)	8.7 (range − 20~30)	29.7 (range 5~55)	< 0.01
Flexion (°)	1.3 (rang −20~20)	12.0 (range 0~35)	0.02
Supination (°)	22.0 (range 0~50)	24.0 (range 0~50)	0.624
Pronation (°)	18.0 (range − 5~40)	20.7 (range 10~40)	0.635
Mayo score	47.3 (rang 40~65)	51.7 (range 25~60)	0.03
MSTS score	17.1 (range 5.7~28.6)	28.6 (range − 2.9~57.1)	< 0.01
VAS score	4.3 (range 2~6)	4.2 (range 1~6)	0.870
Grip strength (% of normal side) (%)	25.1 (range 10.0~47.4)	31.2 (range 11.4~81.6)	0.250

With regards to the variations of functional outcomes before and after surgery, although the prosthesis group showed an improvement in of ROM in the extension, flexion pronation and supination, only the extension (8.7 vs 29.7, *P* < 0.01) and flexion (1.3 vs 12.0, *P* = 0.020) were statistically significant. There was a significant difference in the variation of the Mayo wrist score (47.3 vs 51.7, *P* = 0.03) and the MSTS score (17.1% vs 28.6%, P < 0.01) in two groups. There was no significant difference between the allograft and prosthesis groups in terms of grip strength (25.1% vs 31.2%, *P* = 0.25) and VAS score (4.3 vs 4.2, *P* = 0.87) (Table 4).

With regards to the variations of satisfaction before and after surgery, there was no significant difference in the variation of satisfaction in the Mayo wrist score (20.9 vs 21.6, *P* = 0.726) between the two groups. In addition, there was a significant increase in the variation of emotional acceptance in the MSTS score (4.1 vs 1.4, *P* < 0.01) in the prosthesis group. There was a significantly lower pain reported in the prosthesis group with both the Mayo (25.00 vs 21.88, P < 0.01) and MSTS (5.00 vs 4.38, *P* = 0.02) scores.

### Complications

In the allograft group, all patients were alive, of the 15 patients, only one had a late infection (50 months after surgery), which was caused by plate exposure (type 1B), resulting in debridement and removal of the plate. Four patients had wrist subluxation (type 1A) that occurred 2, 3, 6 and 36 months after surgery. At the lastest follow-up, all patients had degenerative changes (three had grade 2, fourteen had grade 3, mean, 9 months; range 3–12 months), according to the Knirk and Jupiter scale [25]. One patient had resorption of the allograft (type 2B) without allograft bone fracture. None of the included patients had structural failures, soft-tissue failures, nonunion, metastasis, or pain.

In the prosthesis group, none of the patients died; of 15 patients, three patients developed wrist subluxation (type 1A) that occurred within 1 month after surgery. Three patients had separation of the distal radioulnar joint; two of the three patients which occurred in 1 month after surgery, while the other developed within 7 months of the surgery. None of the included patients had structural failures, soft-tissue failures, aseptic loosening, infection, pain, or degenerative changes because of the surgery. There was no significant difference between the allograft and prosthesis group with regards to complications according to the Henderson classification.

**Fig. 3:**
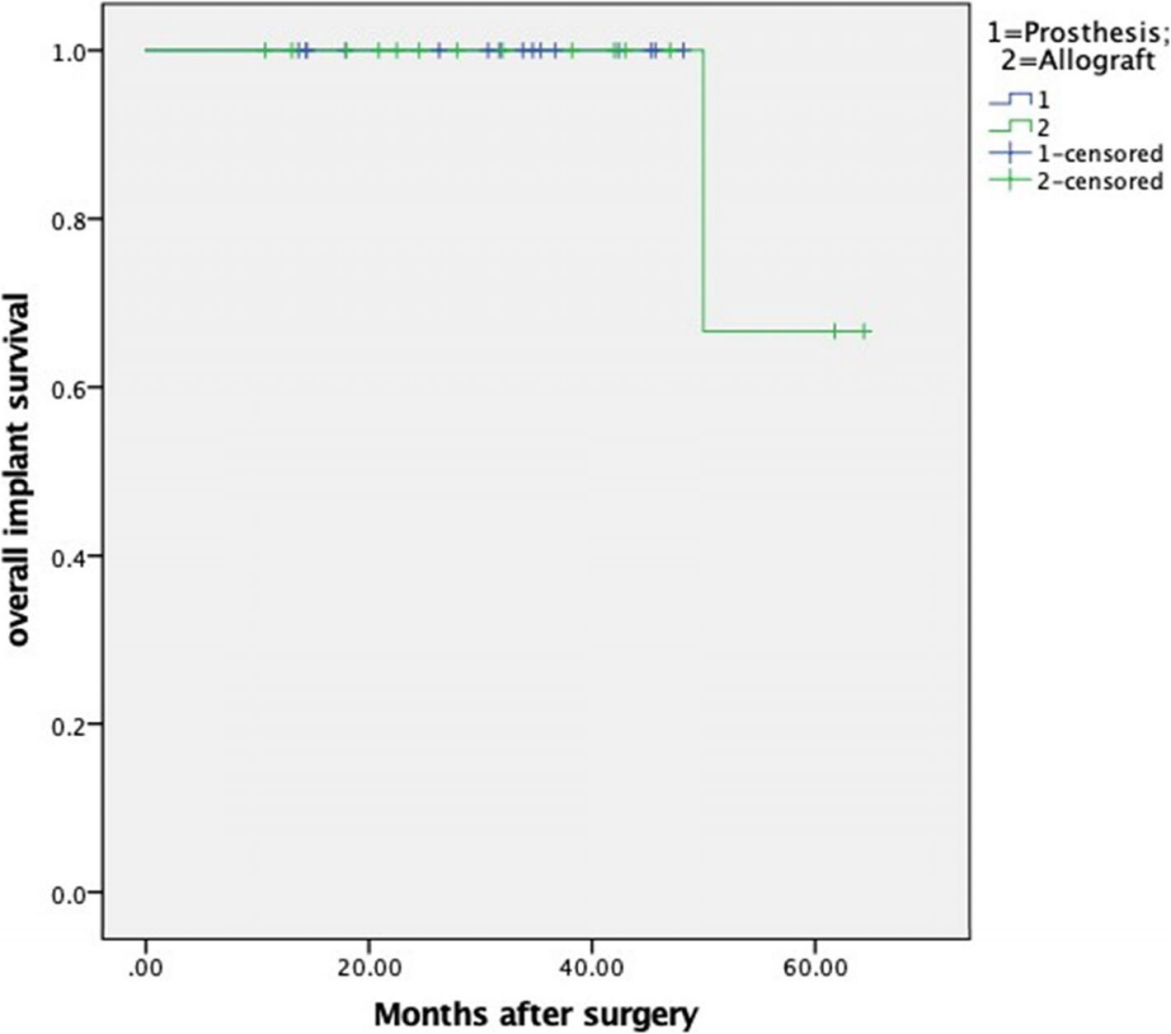
The Kaplan-Meier curve shows the overall implant survival for allograft and prosthesis group, respectively

There was no significant difference between the allograft and prosthesis group in terms of implant survival (*P* = 0.98) (Fig. 3), and the median survival time was not reached in either of the groups. Finally, no patients had died by the time of the last follow-up.

## Discussion

Campanacci III and/or recurrent GCT in the distal radius are characterized by strong invasion and a high recurrence rate. The primary goal of treatment is an oncologic cure. and further functional satisfaction is intensely demanded. The present study is the first to comparing allograft and prosthesis reconstruction for the treatment of GCTs in the distal radius. To highlight differences between the 3D-printed prosthesis and allograft reconstruction, the comparison was performed with regards to functional outcomes and complications. With respect to functional outcome, the MSTS and Mayo score were evaluated; in general, the prosthetic group had a significantly higher scores when compared to the variation of before and after surgery (17.1% vs 28.6%, *P* < 0.01 and 47.3 vs 51.7, *P* = 0.03). In addition, the prosthetic group had a significantly higher score in both MSTS and Mayo, compared to the postoperative evaluation (Table 4).

### Comparison of range of motion (ROM)

For the variation of ROM in the Mayo score, there was a significantly higher score in the prosthesis group compared to the allograft group (4.0 vs 6.4, *P* = 0.04). There was no significant difference between groups with regards to the variation of ROM in the MSTS score (0.67 vs 0.60, *P* = 0.84). This discrepancy in ROM between the Mayo and MSTS scores may be partially explained by the fact that ROM is given a weighting of 25% in the Mayo score compared to 14% in the MSTS system. In addition, most patients received full marks in the MSTS system because the ROM was more than 120 °, and included extension, flexion, supination, pronation, and radial and ulnar deviation. However, the ROM score is valued by a percentage of the contralateral side in the Mayo system, which rarely receives gets full marks.

**Fig. 4:**
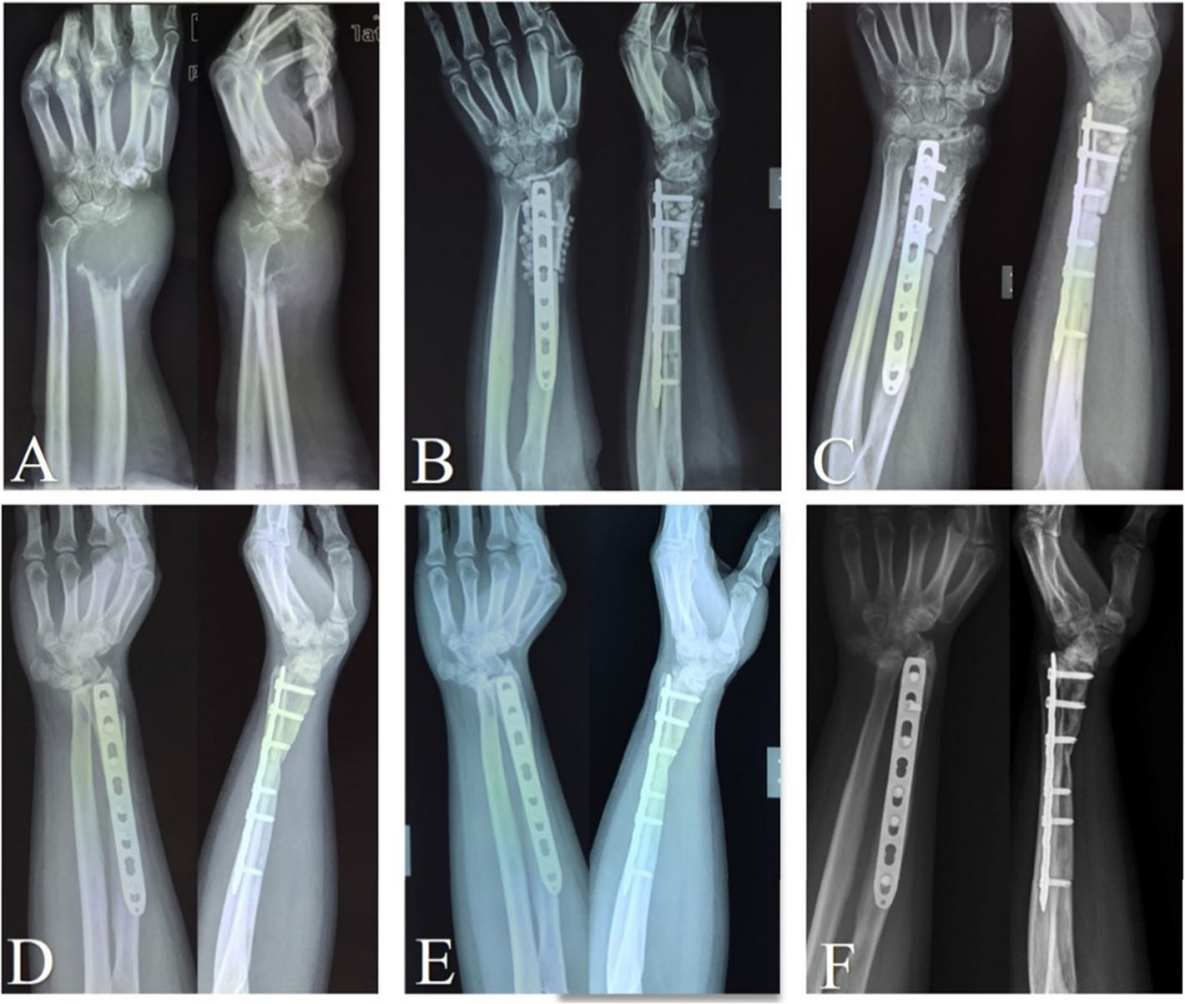
The serial X-rays show the nature of radiological degeneration with allograft reconstruction. **A:** before surgery; **B:** 2 days after surgery; **C:** 2 months after surgery; **D:** 10 months after surgery; **E:** 24 months after surgery; **F:** 54 months after surgery

**Fig. 5:**
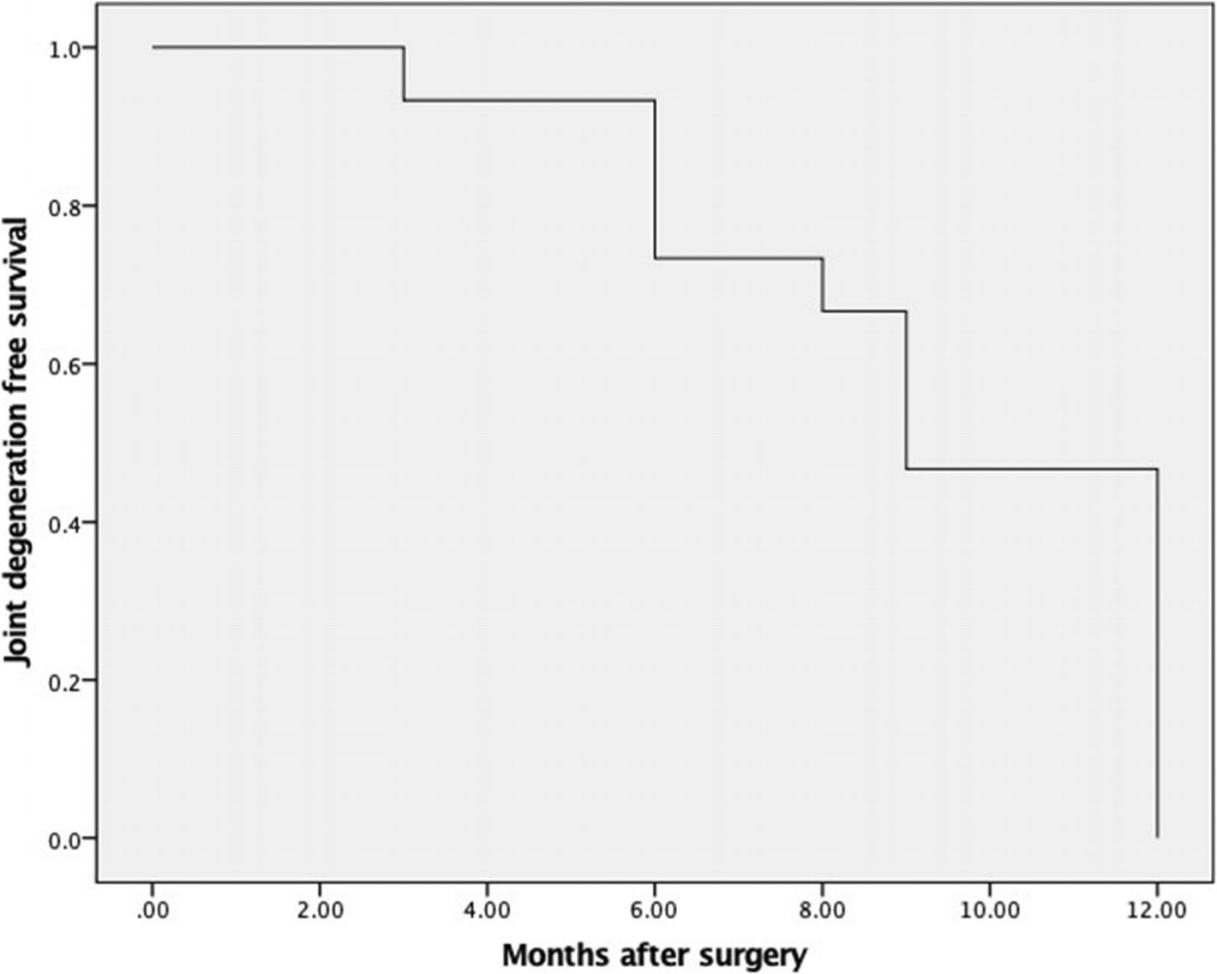
The Kaplan-Meier curves show that the median degeneration-time of wrist was 9 months (95% CI: 8.03–9.97), in all patients with allograft reconstruction after en bloc excision

**Fig. 6:**
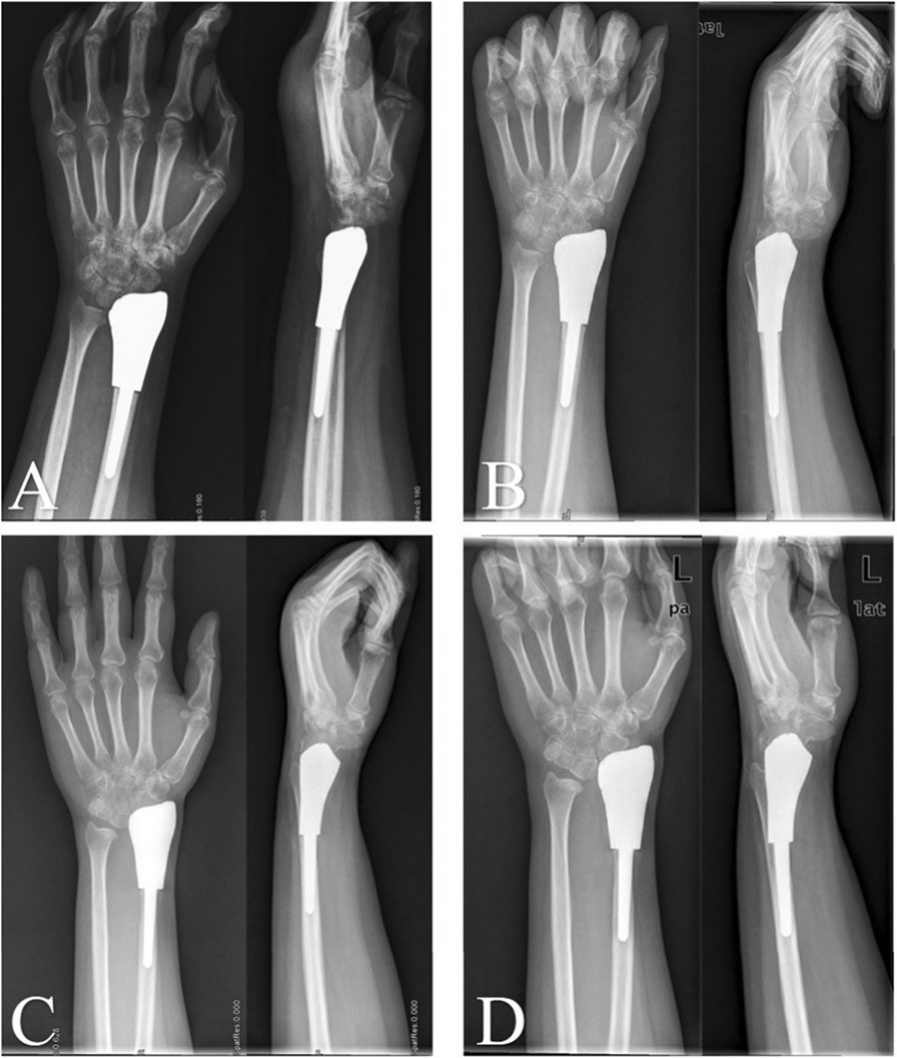
The serial X-rays show the separation of left DRUJ after prosthetic reconstruction. **A:** 2 days after surgery; **B:** 1 month after surgery; **C:** 12 months after surgery; **D:** 42 months after surgery

In previous studies, custom-made cemented prosthesis reconstruction obtained reasonable ROM, with different types, including distal radial prosthesis [9, 11, 20], and total wrist joint prosthesis prothesis [22, 27, 28]. For our 3D-printed uncemented prosthesis, not only the individual and precise design, but also the “press-fit” fixation make surgery easy and result in considerable functional outcomes [21]. Our 3D-printed prosthesis has three main advantages. First, a thick and suitable polyethylene liner is made according to the contralateral side; second, the reserved bone crest of the distal radius on the shaft ensure appropriate implantation without any rotation; and third, seven or eight pores, on the distal prosthetic edge, provide a sufficient area for soft tissue reconstruction. For the allograft group, all patients developed grade 2 or 3 degeneration of the wrist joint (Fig. 4), and the median degeneration-time was 9 months (95% CI: 8.03–9.97) (Fig. 5). There might be an explanation in that creeping substitution, the process through which the allograft is gradually replaced by living bone [29], goes to tide mark under dead articular cartilage, therefore there is a risk of subchondral collapse [30]. With respect to forearm rotation, the distal radioulnar joint (DRUJ) plays a critical role. We reveal that four patients developed separation of the DRUJ (Fig. 6) in prosthetic reconstruction. Based on the tumor border, most structures of the triangular fibrocartilage complex (TFCC) were not preserved in the four patients; therefore, there was a tendency for separation of the DRUJ. With respect to the stabilizing structures of the DRUJ, which includes the TFCC, surrounding ligament, tendon, muscle, interosseous membrane, the bone itself, and the capsule [31]. The TFCC, containing superficial and deep fibers, is the main stabilizer of the DRUJ [32]. Many studies concluded that the dorsal superficial fibers tighten in pronation, as do the deep palmar fibers and vice versa [31]. Therefore, we speculate that the relative decrease in pronation and supination is associated with insufficient reconstruction of the TFCC. When soft tissue reconstruction is achieved, we suggest that the retained fibers of the TFCC should be precisely reconstructed by suturing. No separation of the DRUJ was detected in allograft patients, because of selection bias and longer immobilization postoperatively.

### Comparison of pain

Compared to the postoperative pain, there was a significantly lower level of pain in the prosthesis group in terms of both Mayo (22.3 vs 15.3, *P* < 0.01) and MSTS (5.30 vs 3.13, *P* = 0.04) score. However, the postoperative pain score was not significantly different with VAS evaluation (1.2 vs 1.3, *P* = 0.985). Although the pain was fairly decreased postoperatively in both reconstruction methods, the patients in the prosthesis group complained less of pain compared to those in the allograft group. We speculate that the anatomical and precise design increases the matching of joint compatibility and improved comfort for patients. For prosthesis reconstruction, Zhang et al. reported that only 1 patient complained of moderate pain in a total of 11 patients [9], Wang et al. reported that no patients suffered pain with activity in a total of 10 patients [20]. Secondly, allograft reconstruction has shown a high rate of joint degeneration, which increased the level of pain and decreased the ROM. Rabitsch et al. reported 100% joint degeneration in 4 patients [33], while Duan et al. reported 100% joint degeneration in 15 patients [13].

### Comparison of satisfaction

With respect to postoperative satisfaction in terms of MSTS score, there was a significantly higher satisfaction in the prosthesis group compared to allograft group (2.88 vs 4.25, *P* < 0.01). Nevertheless, there was no significant difference between groups 22.19 vs 23.44, *P* = 0.30) with regards to the Mayo score. This discrepancy may be partially explained by the difference in the weighting of satisfaction in the Mayo and MSTS scores. Although the discrepancy was found in the Mayo and MSTS scores evaluating functional outcomes, a comprehensive comparison was performed between groups using the Mayo and MSTS score. Overall, the prosthetic reconstruction had a better functional outcome, compared to the allograft reconstruction.

### Comparison of complications with Henderson classification

**Fig. 7:**
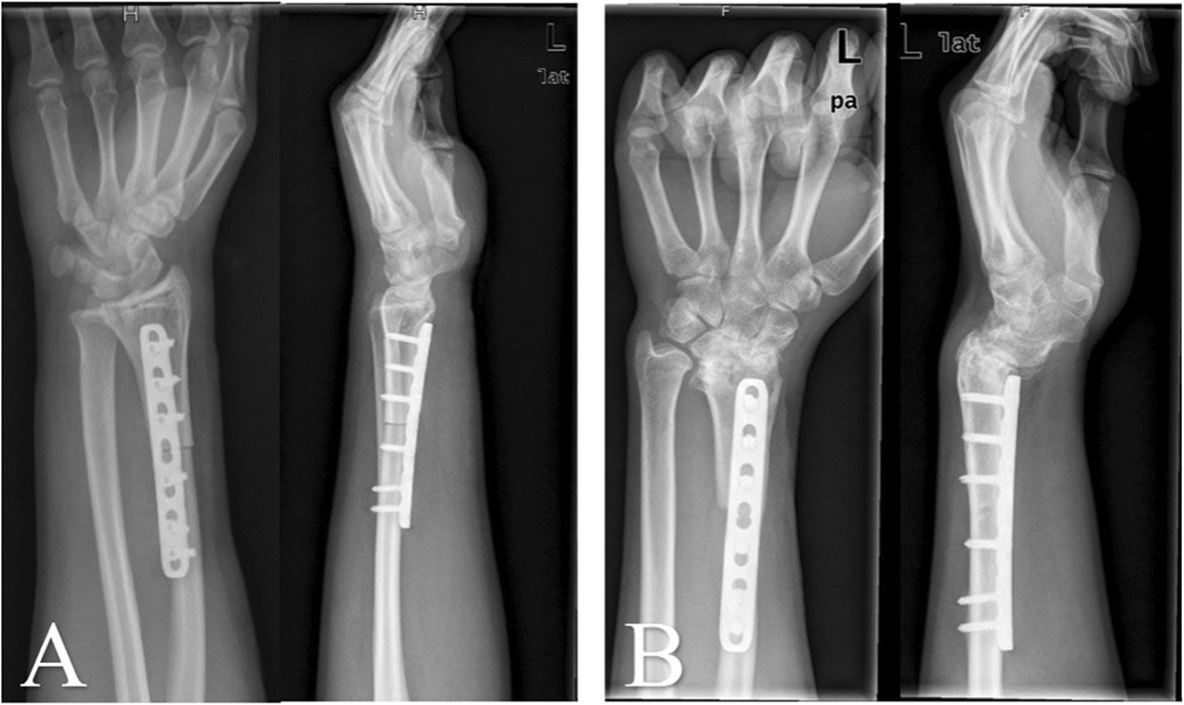
The left palmer subluxation was found in the third year after allograft reconstruction. **A**: 2 days after surgery; **B**: 36 months after surgery

With respect to complications, the main potential problems for the allograft, including nonunion, allograft fracture, wrist osteoarthritis, slow incorporation of the allograft, and rejection, have been reported after en bloc resection (Table 5). Indeed, Bus et al’s compared the complication rates of allograft reconstruction between different sites, and demonstrated that the distal radius showed a significantly lower risk in structural failure and infection compared to the proximal tibia, distal femur, and proximal humerus [38]. Furthermore, the LCP makes reconstructions easy and may be expected to result in fewer complications [13]. As a result, previous authors have suggested that if an intercalary allograft survives the critical 3 to 4 years, it is likely to last for many years [39]. In our study, four patients had palmer subluxation, three of which developed palmer subluxation within the 6 months after surgery. There are three potential reasons for this finding: firstly, the strength of the flexor is greater than that of the extensor [31], developing the tendency of palmer dislocation; secondly, all the patients underwent a dorsal approach, protecting most of the stabilizing structures in the palmar; and thirdly, without the pores in the prosthesis, the retained soft tissue suturing is tedious and unreliable. One patient progressively acquired palmer subluxation in the third year after operation (Fig. 7). According to the radiograph, we speculate that the subluxation was subordinate to the carpal degeneration.

**Table 5:** Summary of the most important published studies on osteoarticular allograft of GCT in the distal radius

Author (year)	Patients (n)	Follow-up (months)	Grip strength	Range of motion	Functional scores	Oncologic Results	Degenerative change	complications
Richard et al. [34](1977)	3	Mean 20.7 (range,6–33)	Mean 35% ^a^	Extension: mean 26.7° Flexion: mean 61.7° Pronation: mean 70° Supination: mean 71.7°	NA.	continuous disease free	NA	Subluxation (1)
Cheng et al. [35](2001)	4	Mean 60 (range,36–96)	Mean 70.3% ^a^	Mean 70% ^a^	MSTS: excellent (3); good (1)	continuous disease free	OA^c^ of the radiocarpal joint (2)	Radioulnar diastasis (2); ulnar translation of carpus (2)
Bianchi et al. [12](2005)	9	Mean 57.3 (range,26–145)	NA	Extension: mean 35.5° Flexion: mean 47.7°	Functional outcome (%)^a^: mean 91.2	Local recurrence (3); lung metastasis (2)	All developed radiographic narrowing	Moderate pain during daily activities (1); ulnar subluxation (5)
Szabo et al. [19](2006)	9	Mean 100 (range,39–219)	Mean 77% ^a^	Extension: mean 52° Flexion: mean 50° Pronation: mean 80° Supination: mean 67°	DASH: mean 15; SF-36: mean 72; Mayo: mean 73	continuous disease free	All developed radiographic narrowing	Minor infection (1); fixation failure (1); flexor carpi radialis tendonitis (1); stress fracture of the allograft (1); ulnar synostoses (2)
Asavamongkolkul et al. [36](2009)	8	Mean 52.7 (range,40.5–90.9)	Mean 72.1% ^a^	Mean 72.5% ^a^	MSTS: mean 93%	Lung metastasis (1)	OA^c^ of the radiocarpal joint (2)	Nonunion (2); graft fracture (1); ulnar translation of carpus (1)
Duan et al. [13](2013)	15	Mean 62.4 (range,36–139)	Mean 27 hg	Extension: mean 46.7° Flexion: mean 33.3° Pronation: mean 72.3° Supination: mean 61.3°	SF-36: mean 71; Mayo: mean 70	Soft tissues recurrence (1)	All developed radiographic narrowing	No
Rabitsch et al. [33](2013)	4	Mean 32 (range,4–121)	Return to prior work	Extension/flexion: mean 60°/38°; Pronation/supination: mean 77°/77°	Mayo: mean 84; DASH: mean 8;	continuous disease free	All patients but no pain	Nonunion (1)
Li et al. [37](2015)	17	Mean 84.7 (range,42–131)	Mean 63.7%^a^	Extension+Flexion (%)^a^: mean 43.5 Pronation+Supination (%)^a^: mean 56.2	MSTS: mean 75%	Local recurrence (1)	Severe (5); moderate (9); minor (3)	Subluxation (3); nonunion (1); avulsion rupture of tendon (2); revised with another allograft (1);
Wysocki et al. [4](2015)	4	Mean 245	Mean 69.4%^a^	Extension: mean 40° Flexion: mean 67.5° Pronation: mean 75° Supination: mean 52.5°	DASH: mean 20; MSTS: mean 87%	continuous disease free	Grade II (2) arthrosis^d^	Nonunion (1)

^a^% of contralateral side; NA, not applicable; ^c^osteoarthritis; ^d^the grading scale of Knirk and Jupiter

**Fig. 8:**
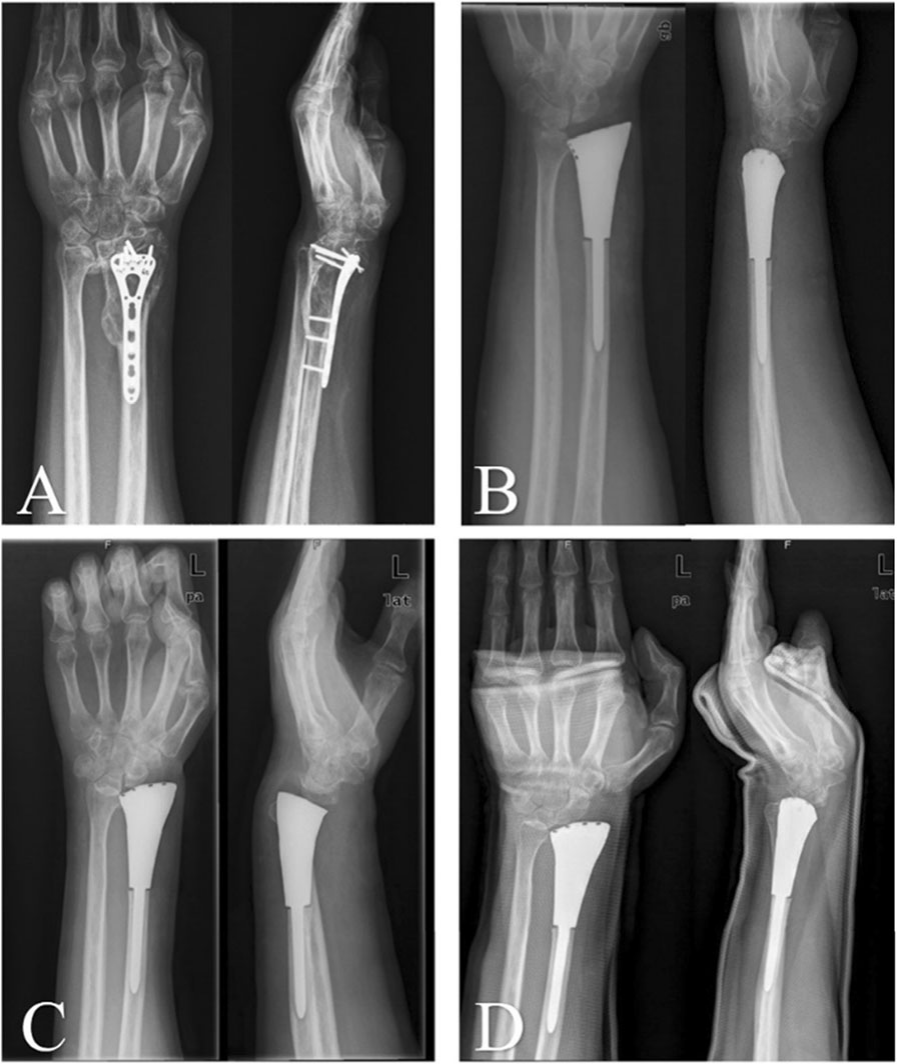
**A**: the recurrent GCT was diagnosed in the left distal radius; **B**: 2 days after prosthetic reconstruction; **C**: the palmer subluxation was found in first month after surgery; **D**: palmer subluxation was not reduced after four weeks immobilization

The main potential complications associated with prosthesis are subluxation, aseptic loosening, infection, soft-tissue failure and fracture of the prosthesis [9, 11, 20, 22, 40] (Table 6). In the present study, three patients developed palmar subluxation that occurred within 1 month of surgery (Fig. 8). In theory, mirroring of the contralateral normal distal radius is the best anatomical match. Accounting for distal radial volar palmar tilt 11–12 °[31], the tendency, for volar subluxation for the carpus to slide off the distal radius, is inevitable. However, soft-tissue tension is affected by the expansive growth of the lesion, especially in Campanacci III or recurrent GCTs of bones [42], and the degeneration of proximal row carpal is generally detected in our elderly patients or those with recurring disease. Hence, total mirroring of the contralateral side may be misleading in this respect, and we propose that the degeneration of the proximal row carpal should be religiously considered. Additionally, it is advisable to sequentially reconstruct retained soft tissue for appropriate soft tissue tension [21]. In addition, radius lengthening combined with folding-plasty of soft-tissue reconstruction is an effective method.

**Table 6:** Summary of the most important published studies on prosthetic replacement of GCT in the distal radius

Author (year)	Patients (n)	Follow-up (months)	Prosthesis design	Device for soft tissue restoration	Grip strength	Range of motion	Functional scores	Oncologic results	Degenerative change	complications
Gold et al. [10](1957)	1	59	Cemented stem	NA	Sufficient for heavy work	A small range of motion	NA	continuous disease free	NA	Fracture of the prosthesis
Hatano et al. [11](2006)	1	168	Cemented stem	purpose-made holes	71% ^a^	Extension: 30° Flexion: 15° Pronation: 30° Supination: 45°	Enneking scale: 83%	continuous disease free	NA	NA
Gokaraju et al. [41](2009)	1	56	Cemented stem	3 mm purpose-made holes	equal to the contralateral side	Extension: 40° Flexion: 20° Pronation: full Supination: 45°	Full DASH: 10.3/100	continuous disease free	Mild	ulna translation of carpus
Natarajan et al. [22](2009)	16	Mean 78 (range, 24–156)	bipolar hinge component with cemented stem	NA	NA	Extension: 20° Flexion: 25° Pronation: 60° Supination: 40°	MSTS: mean 74%	NA	–	Aseptic loosening (2); wound infection (2); skin flap necrosis (2)
Damert et al. [28](2013)	1	24	Cemented stem	NA	34.8% ^a^	Extension: 45° Flexion: 10° Pronation: 80° Supination: 10°	DASH: 25	continuous disease free	NA	No
Hariri et al. [27](2013)	1	33	Cemented stem	NA	63% ^a^	Extension: 70° Flexion: 20° Pronation: 70° Supination: 60°	Quick DASH: 52.3/100; Enneking scale: 83%	continuous disease free	NA	No
Zhang et al. [9](2015)	11	Mean 55.5 (range, 24–83)	Cemented stem	purpose-made holes	Mean 33.1%^a^	Extension: mean 40.1° Flexion: mean 30° Pronation: mean 38.2° Supination: mean 46.4°	MSTS: mean 80%	continuous disease free	No	Superficial infection (1); pain (1)
Wang et al. [20](2016)	10	Mean 52 (range, 24–90)	Cemented stem	purpose-made holes	Mean 68% ^a^	Extension: mean 40.1° Flexion: mean 30° Pronation: mean 38.2° Supination: mean 46.4°	Mayo: mean 68	continuous disease free	Grade 0 (7); grade 1 (2); grade 2 (1) arthrosis^b^	Aseptic loosing (1); pain (2)

NA, not applicable; ^a^% of contralateral side; ^b^the grading scale of Knirk and Jupiter

Most studies report wrist joint degeneration after allograft reconstruction (Table 5), while wrist joint degeneration was rarely detected using the prosthesis method. Duan et al. reported that degeneration was revealed in a mean of 4 months postoperatively [13], while we found degeneration with a mean of 9 months. We speculate that the wrist joint degeneration secondary to allograft reconstruction develops in the first year postoperatively. With respect to the comparison of wrist subluxation, although there was no significant difference between the allograft (4 patients) and prosthesis (3 patients) groups, different mechanisms were found in the two groups. For allograft reconstruction, subluxation was subordinate to the gradual carpal degeneration, while subluxation was mostly dependent on the prosthetic design and retained soft tissue.

This study presents several limitations, mainly due to the nature of the disease. Firstly, our study was retrospective with a small size (15 patients in each group). As such, our small sample size may be expected to result in low statistical power. Secondly, the follow-up time was significantly different in the two groups, and the follow-up time is insufficient to make conclusions on the long-term implications of the result. Thirdly, no patient was administered denosumab preoperatively. The efficacy of denosumab has been demonstrated in patients with unresectable or recurrent GCT of bone, according to the NCCN guideline and previous studies [43]. However, denosumab did not show any effect on reducing the recurrence rate [44], and complications such as sarcomatous transformation should be considered [45]. Fourthly, our findings are only based on the respective data from our institution, this implies a study selection bias that must be acknowledged, and which might only reflect surgeon or patient preference. As such, this may have had a substantial impact on our observations. Finally, we did not have sufficient reconstruction types such as autograft fibula grafts, for arthrodesis or osteoarticular reconstructions nor did we look at vascularized fibular grafts; as a result, our ability to state that arthrodesis is a superior reconstruction method is limited and we can only show that the results in our patients provided them with reasonable function.

## Conclusions

This is the first study comparing the objective functional outcomes and complications of two reconstructive methods for the Campanacci III or recurrent GCTs in the distal radius. Despite including subluxation cases, 3D-printed prosthesis replacement at short-term follow-up can partially preserve better wrist function than osteoarticular allograft reconstruction at short-term and even median-term follow-up. During the 3D-printed prosthesis design, preoperative morphological assessment of the affected proximal row carpal is helpful to control for postoperative dislocation. In addition, considering the relative instability of joint capsule reconstruction, properly delayed rehabilitation is recommended. After osteoarticular allograft reconstruction, wrist degeneration, which has been proven in all patients, severely influence their wrist function compared to the patients with prosthesis reconstruction. Therefore, compared to osteoarticular allograft reconstruction, 3D-printed prosthesis reconstruction has its irreplaceable advantages at early-stage application, especially in terms of reconstruction of the wrist function, although further study of cases with follow-up is necessary.

## Data Availability

The datasets used and analyzed during the current study are available from the corresponding author on reasonable request.

## Abbreviations

3D: Three-dimensional
DRUJ: Distal radioulnar joint
GCT: Giant cell tumor
MSTS: Musculoskeletal tumor society
ROM: Range of motion
TFCC: Triangular fibrocartilage complex
